# Late Recurrence of Pancreatic Solid Pseudopapillary Neoplasm With Peritoneal Carcinomatosis Treated With Cytoreductive Surgery (CRS) and Hyperthermic Intraperitoneal Chemotherapy (HIPEC): A Case Report

**DOI:** 10.7759/cureus.31189

**Published:** 2022-11-07

**Authors:** Jorge Nogueiro, Fábio Gomes, João Pacheco, Hugo Santos-Sousa, Sara Meireles, Renato Bessa Melo, Marisa Aral, Elisabete Barbosa

**Affiliations:** 1 General Surgery, Centro Hospitalar Universitário de São João, Porto, PRT; 2 Faculty of Medicine, Universidade do Porto, Porto, PRT; 3 Anatomical Pathology, Centro Hospitalar Universitário de São João, Porto, PRT; 4 Oncology, Centro Hospitalar Universitário de São João, Porto, PRT; 5 General Surgery, Centro Hospitalar Universitário Cova da Beira, Covilhã, PRT

**Keywords:** late recurrence, peritoneal metastasis, hyperthermic intraperitoneal chemotherapy, cytoreduction surgical procedures, pancreatic neoplasms

## Abstract

Pancreatic solid pseudopapillary neoplasm (SPN) is a rare malignant tumour predominantly affecting young women. The occurrence of peritoneal carcinomatosis (PC) in this setting is an even rarer condition, usually related to perioperative tumour rupture. We present a case of a 43-year-old woman who previously underwent distal splenopancreatectomy after the diagnosis of a pancreatic SPN. Thirteen years later, the patient underwent a radical hysterectomy due to a uterine myoma. Intraoperatively, a peritoneal mass was additionally found and resected. Histological examination revealed an implant with morphology compatible with pancreatic SPN. The patient was then referred to our institution. Staging MRI and CT revealed multiple nodular lesions adjacent to the left colon, suggestive of peritoneal implants. The patient was then submitted to cytoreductive surgery (CRS) combined with hyperthermic intraperitoneal chemotherapy (HIPEC) with oxaliplatin and irinotecan. Histological examination confirmed peritoneal involvement by a pancreatic SPN. The postoperative course was unremarkable. Two years after surgery, the patient remains asymptomatic with no evidence of relapse. Despite SPN being cancer with a relatively indolent evolution, one needs to be aware of a possible recurrence several years after the primary resection, mainly in patients with evidence of intraoperative tumour rupture.

## Introduction

Solid pseudopapillary neoplasm (SPN) of the pancreas is a rare malignant tumour, comprising only 0.13% to 2.7% of all pancreatic neoplasms [1,2]. It predominantly affects young women between 30 to 40 years of age, with a female/male ratio of 8.25-9.78/1 [1,3]. Most patients are diagnosed at an early stage, abdominal pain being the most common complaint [1,3]. Distant metastases occur in 10-15% of patients [2], but peritoneal carcinomatosis (PC) has been reported in only 12 cases [2,4-14]. Although early-stage disease and even locally advanced or metastatic disease are often amenable to complete surgical resection and are associated with long-term survival, the approach to PC is not well established, frequently with disappointing results, even with complete cytoreductive surgery (CCRS) [2]. We present a case of a late recurrence of pancreatic SPN with PC treated with CCRS and hyperthermic intraperitoneal chemotherapy (HIPEC).

This case was previously presented as a meeting abstract/poster at the 40th Congress of the European Society of Surgical Oncology in Lisbon from November 8-10, 2021.

## Case presentation

**Figure 1:**
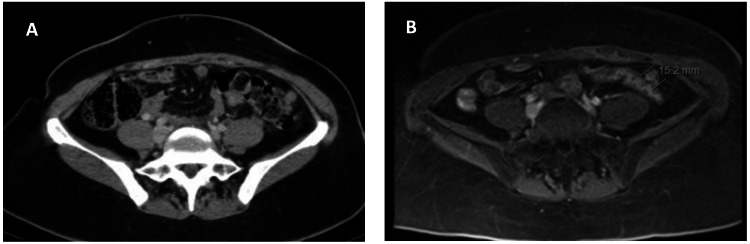
Preoperative CT (A) and MRI (B) scans revealing metastatic peritoneal nodules adjacent to the left colon

We present a case of a 43-year-old woman previously submitted to distal splenopancreatectomy when she was 30, following the diagnosis of a pancreatic tail SPN in another institution. Thirteen years after the surgery, the patient was submitted to a radical hysterectomy due to a uterine myoma. Intraoperatively, a peritoneal mass of 2.4 x 2.0 cm was incidentally found and resected. Histological examination revealed an implant with morphology compatible with the peritoneal recurrence of a pancreatic SPN. The patient was then referred to our institution. She had no remarkable medical history besides arterial hypertension and type 2 diabetes mellitus. Staging magnetic resonance imaging (MRI) and computed tomography (CT) revealed multiple nodules adjacent to the left colon, suggestive of peritoneal implants (Figure 1)

**Figure 2:**
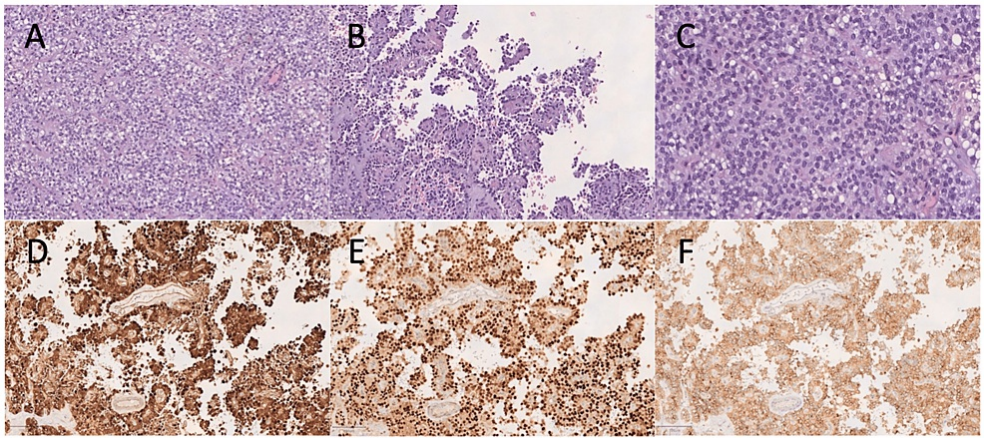
Histological appearance of one of the neoplastic implants in the great omentum Areas of solid (H&E, 100x) (A) and pseudopapillary patterns (H&E, 100x) (B) were seen, with associated fibrovascular stalks containing variable degree of myxoid change and hyalinization. Cells displayed ovoid nuclei with fine chromatin and characteristic longitudinal grooves; the cytoplasm was occasionally vacuolated and exhibited hyaline globules (H&E, 200x) (C). Nuclear and cytoplasmic expression of β-catenin was demonstrated by immunohistochemistry (100x) (D). Additionally, expression of cyclin D1 (100x) (E) and CD10 (100x) (F) was observed. Synaptophysin was expressed focally, whereas chromogranin expression was absent (not shown). H&E: hematoxylin and eosin stain

Intraoperatively, the peritoneal carcinomatosis index was 5. The patient was then submitted to CCRS combined with HIPEC with oxaliplatin (360mg/m2) and irinotecan (360mg/m2) at a temperature of 43ºC for 30 min. Histological examination confirmed the involvement of the great omentum, right diaphragmatic peritoneum and multiple peritoneal fragments by a pancreatic SPN (Figure 2).

The postoperative course was unremarkable, and no adjuvant chemotherapy was administered. The patient remains asymptomatic with no evidence of relapse at two years follow-up.

## Discussion

Pancreatic SPN is a rare malignant tumour, and only 12 cases were reported in the literature with progression to PC during the natural history of the disease [2,4-14]. Tumour spillage in the context of intra-operative tumour rupture has been associated with peritoneal recurrence [2]. Data concerning the initial distal splenopancreatectomy in the current case performed in another institution are not available. Peritoneal recurrence after tumour rupture may occur 1 to 19 years after the primary surgery [2], which may raise the issue of a longer follow-up time than other cancer types typically occurring in the gastrointestinal tract, especially in the pancreas.

Since it is a relatively indolent tumour, surgery yields very good long-term results with five-year overall survival (OS) of 93.4-100%, even in the presence of locally advanced or metastatic disease [2,15,16]. Locoregional recurrence occurred in 100% of cases after an R2 resection or tumour rupture [17]. Neoadjuvant treatments have been used to shrink the tumour and avoid intra-operative spillage but their role remains unclear [4]. All these features should prompt an aggressive primary surgical approach to achieve the best oncological results.

Patients with PC from pancreatic SPN treated with surgery alone had disappointing results, with a peritoneal recurrence rate of 58% (7/12). The real recurrence rate might be underestimated owing to the short follow-up time reported in the literature. Nevertheless, none of these patients developed other distant metastasis.

We decided to add HIPEC with oxaliplatin and irinotecan similar to the approach previously described, and after a 24-months follow-up, the patient displays no signs of recurrence [2].

We need to have a longer follow-up to confirm that performing a CCRS-HIPEC is a valid option for patients with pancreatic SPN PC. Nevertheless, the two patients treated with this modality in the literature remain alive, with no signs of recurrence [2].

## Conclusions

In spite of the indolent evolution of SPN, there should be a close follow-up, considering a possible recurrence several years later, as it can give rise to extensive peritoneal disease. A well-performed first surgical excision, without tumour spillage, is crucial to avoid a peritoneal recurrence. Nonetheless, the surgery-only approach to patients with PC has yielded disappointing results, with high rates of peritoneal recurrence. Despite the scant data in the literature, an approach with CCRS plus HIPEC might be a more efficient approach to treating patients with PC. Nevertheless, this approach should be further validated by prospective trials in order to properly assess the role of CCRS and HIPEC in this group of patients.
